# Assessment of the Benefits and Cost-Effectiveness of Population-Based Breast Cancer Screening in Urban China: A Model-Based Analysis

**DOI:** 10.34172/ijhpm.2021.62

**Published:** 2021-07-04

**Authors:** Jing Wang, Marcel J.W. Greuter, Senshuang Zheng, Daniëlle W.A. van Veldhuizen, Karin M. Vermeulen, Yuan Wang, Wenli Lu, Geertruida H. de Bock

**Affiliations:** ^1^Department of Epidemiology, University Medical Center Groningen, University of Groningen, Groningen, The Netherlands.; ^2^Department of Radiology, University Medical Center Groningen, University of Groningen, Groningen, The Netherlands.; ^3^Robotics and Mechatronics (RaM) Group, Faculty of Electrical Engineering Mathematics and Computer Science, Technical Medical Centre, University of Twente, Enschede, The Netherlands.; ^4^Department of Epidemiology and Health Statistics, School of Public Health, Tianjin Medical University, Tianjin, China.; ^5^Collaborative Innovation Center of Chronic Disease Prevention and Control, School of Public Health, Tianjin Medical University, Tianjin, China.

**Keywords:** Breast Neoplasms, Mammography, Mass Screening, Cost-Benefit Analysis, Urban China

## Abstract

**Background:** To decrease the burden of breast cancer (BC), the Chinese government recently introduced biennial mammography screening for women aged 45-70 years. In this study, we assess the effectiveness and cost-effectiveness of implementing this programme in urban China using a micro-simulation model.

**Methods:** The ‘Simulation Model on radiation Risk and breast cancer Screening’ (SiMRiSc) was applied, with parameters updated based on available data for the Chinese population. The base scenario was biennial mammography screening for women aged 45-70 years, and this was compared to a reference population with no screening. Seven alternative scenarios were then simulated by varying the screening intervals and participant ages. This analysis was conducted from a societal perspective. The discounted incremental cost-effectiveness ratio (ICER) was compared to a threshold of triple the gross domestic product (GDP) per life years gained (LYG), which was 30 785 USD/LYG. Univariate sensitivity analyses were conducted to evaluate model robustness. In addition, a budget impact analysis was performed by comparing biennial screening with no screening at a time horizon of 10 years.

**Results:** Compared with no screening, the base scenario was cost-effective in urban China, giving a discounted average cost-effectiveness ratio (ACER) of 17 309 USD/LYG. The model was most sensitive to the cost of mammography per screen, followed by mean size of self-detected tumours, mammographic breast density and the cumulative lifetime risk of BC. The efficient frontier showed that at a threshold of 30 785 USD/LYG, the base scenario was the optimal scenario with a discounted ICER of 25 261 USD/LYG. Over 10 years, screening would incur a net cost of almost 38.1 million USD for a city with 1 million citizens.

**Conclusion:** Compared to no screening, biennial mammography screening for women aged from 45-70 is cost-effective in urban China.

## Background

**Key Messages**

** Implications for policy makers**

- The results showed that biennial mammography screening was cost-effective in urban China compared with no screening.
- The scenarios analysis indicated that the recommended screening age interval of 45 to 70 is suitable for the urban Chinese women.
- The budget impact analysis showed that during ten years, screening would incur a net cost of US$38.1 million for a city with one million citizens.

** Implications for the public**

Breast cancer (BC) is the most common cancer amongst women in China. To reduce disease burden of BC, in 2019, the Chinese government introduced biennial mammography screening for women aged 45-70. However, it is unknown whether mammography screening could be beneficial and cost-effective in the Chinese women. The results showed that biennial mammography has the potential to reduce BC deaths and to achieve more life year compared with not performing screening. Regarding the screening starting age, scenarios starting from a younger or older age did not contribute to more life years compared to the base scenario (starting from age 40). These findings provide useful evidence on screening cost-effectiveness and are of importance for the optimization of BC screening strategies in China.

Breast cancer (BC) is the most common cancer amongst women in China.^1^ In 2013, newly diagnosed BCs were estimated to account for 17% (278 800) of all new cancers in China,^2^ and over the last few decades, the incidence has increased by an estimated 3% annually.^3^ The age-standardized rate of mortality by world standard population was 6.34 per 100 000 and the absolute number of BC-related deaths was 64 600 in 2013.^2^ A recent study has shown that in 2017, years of life lost due to BC was 169 per 100 000 population.^4^ In urban areas, the age-specific incidence of BC increases dramatically after age 30 years, peaking at a rate of 111.75 per 100 000 by age 55 years.^2^ Compared with Western countries, BC is often diagnosed at a more advanced stage in China, at which point it is more difficult to treat and cure, leading to an increased disease burden on society.^5^

 Though still a matter of debate, regular mammography screening has been shown to have the potential to reduce mortality by detecting cancer at early stages, allowing for more effective treatment to improve survival.^6^ Indeed, a meta-analysis has shown that the introduction of mammography screening programmes can achieve a 20% mortality reduction.^7^ Over recent decades, China has established several large trials of screening, including the urban Chinese National BC Screening Program (CNBCSP-urban) and the CNBCSP-rural, as well as the Multi-modality Independent Screening Trial.^8,9^ In the CNBCSP, women aged 35-69 years were primarily screened by clinical breast examination, with mammography or ultrasound reserved for when abnormalities were found. By contrast, the Multi-modality Independent Screening Trial programme used a combination of all three modalities to screen asymptomatic women aged 45-65 years. The preliminary results of the CNBCSP indicated that screen-detected tumours were of a lower stage and smaller size than those diagnosed in clinical settings,^8^ which suggested that more effective and less-aggressive treatments can be used to improve survival and reduce disease burden. Nevertheless, it remains doubtful whether breast conserving surgery would be widely used in China if factors such as the less accessibility of radiotherapy, and the additional costs due to breast conserving surgery and postoperative radiotherapy were taken into account.^10^

 In 2019, the Chinese government introduced a mammography-based biennial screening strategy for women aged 45-70 years.^11^ However, there are several barriers to population-based mammography screening for BC in China. First, as long-term effects such as mortality reduction have not been proven in Chinese women, more evidence from studies with long term follow-up might be of importance in the evaluation of screening effectiveness.^11^ Second, China has a large population, and such programmes therefore require substantial medical and financial resources.^12^ Third, little has been reported to date on the cost-effectiveness of this approach.^12^

 In this study, we aimed to assess the effectiveness and cost-effectiveness of implementing a biennial mammography screening programme for Chinese women. Given that evaluating the effects and cost-effectiveness of BC screening needs a long follow-up time and a large population, we opted to employ a micro-simulation model to help evaluate these and provide some early evidence to guide the implementation of an optimal screening strategy in an economical way. Additionally, marked disparities have been reported between urban and rural populations in the incidence and survival related to BC. Thus, we focused on urban Chinese women because there is a relative shortage of mammography equipment in rural China and because mammography is more accessible in urban areas.

## Methods

 This study was reported according to the Consolidated Health Economic Evaluation Reporting Standards (CHEERS) statement.^13^ The Simulation Model on radiation Risk and breast cancer Screening (SiMRiSc) was applied in the current analysis,^14-18^ with parameters updated based on currently available data for the Chinese population.

###  Model Description

 The SiMRiSc model is a micro-simulation model, written in C++, and has previously been used for Caucasian women with *BRCA* mutations or in the general population.^14-18^ In summary, women’s lifetimes were simulated by considering their life expectancy, the chance of developing cancer, tumour growth, tumour self-detection probability and survival probability (from BC). Age-specific mortality in the general population was used to determine the death age for women without BC. If a tumour was detected during screening, the chance of detection depended on mammographic sensitivity, which in turn, was dependent on the percent mammographic density and tumour size.^19^ After diagnosing BC, either by screening or self-detection, the BC age-specific death of a woman was calculated based on expected life expectancy, and this depended on tumour size. The BC survival was modelled as a function of tumour size at diagnosis and years after diagnosis,^20^ a detailed description can be found in Supplementary file 1. Also, mammographic specificity for the introduction of false positives and the probability of tumour induction due to ionising radiation from mammography were included. All primary invasive BCs were modelled in this study, and cancer recurrence was not considered.

 The estimates for the model input parameters were based on population statistics and the results of systematic searches (Table 1).^9,17,19,21-34^ If no studies were found that focused on the Chinese population, studies from other Asian or from Western populations were used. We did not use data from the CNBCSP-urban trial to obtain suitable input parameters for our model, as this trial used clinical breast examination with ultrasound or mammography as the main screening modality, which is different from our modelled screening strategies. Age-specific incidence and mortality rates were obtained from data for the Chinese population^21,22^ (related data is shown in Supplementary files 2 and 3).

**Table 1:** Input Variables and Their Estimates for the SiMRiSc Model

	**Variables**		**Estimates (95% CI)**	**Reference**
Population	Lifetime risk (%)		4.34 (4.18-4.50)	^21^
	Mean onset age		58.76 (58.51-59.02)	
	Spread		16.99 (16.68-17.30)	
	Mortality		Supplementary file 3	^22^
Tumour growth model (TVDT)	Geometric mean of doubling time (log transformed)		5.16 (4.96-5.36)	^23^
	Spread of doubling time		0.98	
Self-detection diameter (cm)	Mean of self-detection size		2.92 (2.84-3.01)	^25^
	Spread of self-detection size		0.66	
Tumour induction	Probability of tumour induction due to radiation per Gy		0.51 (0.28-0.83)	^17^
Mammography	Radiation dose (per screen) in mGy		3.00 (1.00–5.00)	^17^
	Specificity		0.94 (0.90-0.97)	^9,26-34^
	Sensitivity function (Supplementary file 4)	m (%)^b^	18.6 (0-42.9)	^24^
		*β*_1_	-4.38 (-3.76, -3.98)	^19^
		*β*_2_	0.49 (0.40, 0.60)	
		*β*_3_	-1.34 (-3.00, -0.08)	
		*β*_4_	-7.18 (-16.11, -2.77)	
Costs	Mammography (per screen)		$34	^35^
	Biopsy		$43	^35^
	Costs related to treatment^a^	<2 cm	$7 955	^36^
		2-5 cm	$8 074	
		>5 cm	$10 128	

Abbreviations: TVDT, tumour volume doubling time; SiMRiSc, Simulation Model on radiation Risk and breast cancer Screening. ^a^Both medical and non-medical expenditure were included; ^b^m (%) stands for mammographic percentage density.

 For the tumour growth model, exponential tumour growth was assumed. The tumour volume doubling time (TVDT) was assumed to be log-normally distributed with a geometric mean TVDT of 174 days based on data from a Japanese study.^23^ In addition, mammography sensitivity was modelled as a logistic function that depended on tumour size and percent mammographic density.^19^ A detailed description of the sensitivity function is provided in Supplementary file 4. The mean percent mammographic density for screen-detected cancers was 18.6%, based on data from a Korean population.^24^ Because no reliable population-based estimates were available for the specificity of mammography in the Chinese population, we performed a meta-analysis in an Asian population, which revealed a pooled specificity of 0.94 (95% confidence interval [CI]: 0.90-0.97).^9,26-34^ Detailed information related to the meta-analysis can be found in Supplementary file 5.

###  Cost

 The cost-effectiveness analysis was conducted from a societal perspective. The costs of mammography and core needle biopsy were obtained from the Tianjin Development and Reform Commission.^35^ The direct costs related to treatment were derived from the study by Liao et al in which both medical expenditure and non-medical expenditure were included.^36^ For medical expenditure, all medial costs during two months before and ten months after diagnosis were included, both costs covered by health insurance and out-of-pocket money were considered. According to Liao et al, only 45%-50% medical expenditure was covered by the health insurance. Regarding to non-medical expenditure, additional meals, additional nutrition, transportation, accommodation, cost of informal nursing and other out-of-pocket costs were considered. All costs in the model were calculated in United States dollars (USD), with 2019 used as the reference year. The CCEMG-EPPI-Centre Cost Converter (v1.6) was used to adjust costs to USD and for price year.^37^

###  Validation of the Model

 The SiMRiSc model was validated by comparing the model-predicted outcome with published data. Due to limited data on BC screening in Asia, we could only externally validate our model based on three outcomes: the cancer detection rate (CDR), the screen-detected size distribution and the self-detected tumour size distribution. Population-based data from Japan were used for model validation,^38-40^ and the incidence data for Japan is shown in Supplementary file 2, Table S1.^40^ To generate comparable results according to the Japanese BC screening guideline, a biennial screening scenario covering ages 40-74 years was used for validation.^41^ An attendance rate of 18.3% was applied in the simulation based on the Japanese data.^38^

###  Base Scenario

 In accordance with the Chinese BC screening guidelines for 2019, the base scenario was biennial mammography screening for women from their age 45 to 70 years old, and the reference scenario was all women not undergoing screening. Given that participation rate is a key factor that influences the effectiveness and cost-effectiveness of screening programmes, several participation rates were assessed for the base scenario (ie, 100%, 80% and 60%).

###  Outcomes

 We simulated 100 000 women to minimise the risk of statistical error and to limit the computation time. Each simulation was repeated 10 times to calculate the error of the point estimates, and the results were reported in terms of averted tumour deaths, screen-detected tumours, interval cancers and life years gained (LYG) per 100 000 women over their lifetimes. Interval cancers were defined as cancers diagnosed after a negative mammographic screen and before the next scheduled screen in women that participated in the screening. In our analysis, interval cancers were categorized as true interval cancers (cancers that became detectable after the previous screening and were self-detected before the next screening), and missed cancers (cancers that were missed by the previous screening round). Average cost-effectiveness ratios (ACERs) were estimated as the ratios of the additional costs of the screening scenario to the LYG compared to no screening. In addition, incremental cost-effectiveness ratios (ICERs) were calculated based on the comparison of a lower cost scenario to the next more expensive and effective scenario after excluding dominated scenarios. A discount rate of 5% for both costs and health effects (LYG) was applied based on the China Guidelines for Pharmacoeconomic Evaluations.^42^ The willingness-to-pay threshold was estimated as triple the gross domestic product (GDP) per capita in China in 2019,^43,44^ equating to 30 785 USD/LYG. All ICERs are reported as discounted ICERs unless otherwise specified.

###  Alternative Scenario Analyses and Sensitivity Analyses

 Seven alternative scenarios were performed by varying the screening interval (2 or 3 years), screening start age (from age of 40, 45 or 50 years) and stop age (65 or 70 years). We did not expand the screening age to 75 years old as the life expectancy for the urban Chinese women is around 79 years of age.^45^ The robustness of our model was tested using univariate sensitivity analysis. For each model input parameter, univariate sensitivity analysis was performed based on the lower and upper bounds of the 95% CI; For cost inputs, we performed the analysis by varying the costs by ± 50%. Tornado plots were generated to visualise the impact of parameter uncertainty on the screening cost-effectiveness.

###  Budget Impact Analysis

 A budget impact analysis was conducted to evaluate the implementation of biennial mammography screening for women aged 45-70 years. This estimated the net cumulative cost of the screening programme and its costs, such as related biopsy and treatment, for the public healthcare payer over 10 years. We provided cost estimates for a medium-sized city with a population of 1 million citizens rather than estimating the costs for the entire urban population in China. This analysis was repeated with a participation rate of 80%. The age distribution data for the most recent year, 2017, were extracted from the National Bureau of Statistics of China (see Supplementary file 6), and we assumed that 7500 new cases of 45-year-old women would be added to the programme annually.^46^

## Results

###  Validation of the Model

Table 2 shows the comparisons between the observed and simulated outcomes. The simulated CDR was slightly larger than the observed CDR (3.7‰ vs 3.2‰). Notably, the proportion of screen-detected tumours ≤2 cm was underestimated in the simulated (75.8%) compared with the observed (80.4%) data, but the proportions of screen-detected tumours ≤5 cm were comparable (98.0% vs 97.6%, respectively). Although the distribution of self-detected tumour size was comparable between the data sets, tumours ≤2 cm were slightly overestimated in the simulated model.

**Table 2:** The Validation of the Simulation Results

	**Simulated (95%CI)**	**Observed (95% CI)**	**Reference**
Cancer detection rate (‰)	3.7	3.2 (3.1-3.3)	^38^
Tumour size distributions of screen-detected cancers (%)			
≤2 cm	75.8	80.4 (80.0-80.8)	^39^
2–5 cm	22.2	17.3 (16.9-17.7)	
>5 cm	2.0	2.4 (2.2-2.5)	
Tumour size distribution of self-detected cancers (%)			
≤2 cm	57.3	55.9 (55.5-56.2)	^40^
2–5 cm	37.2	38.7 (38.4-39.0)	
>5 cm	5.5	5.3 (5.2-5.5)	

###  Base Scenario

 Biennial mammography screening for 100 000 women aged 45-70 years was estimated to reduce BC deaths by 312 and to achieve 1747 screen-detected cancers and 7963 LYGs compared with not performing screening, assuming a 100% participation rate. In addition, the number of interval cancers was estimated to be 1388, of which 48% were true interval cancers that were not missed at the previous screening round. True interval cancers grew faster (median TVDT: 67 days) than screen-detected cancers (median TVDT: 251 days) and interval cancers that were missed at the previous screening (mean TVDT: 160 days). Forty cancers were considered to be overdiagnosed in the base scenario (Supplementary file 7, Table S6). When the participation rate decreased, fewer averted BC deaths, screen-detected cancers, radiation-induced tumours and LYGs were estimated (Table 3). The discounted ACER was 17 309 USD/LYG at a participation rate of 100%. Although the ACER became slightly more favourable at a lower participation rate, this was at the expense of a large decrease in averted deaths, screen-detected cancers and LYGs.

**Table 3:** Modelled Cost-Effectiveness of Different Scenarios Compared to No Screening

**Scenarios**	**Averted BC Deaths**	**Screen-Detected Cancers**	**Radiation-Induced Tumour**	**Interval Cancers**	**LYG**	**Total Cost ** **($, Million)**	**Discounted LYG** ^a^	**ACER ($/LYG)**	**Discounted ACER** ^a^ **($/LYG)**
Base scenario	312 (6)	1 747 (15)	64 (2)	1 388 (12)	7 963 (156)	79.1 (0.1)	1 670 (39)	5 807 (106)	17 309 (363)
Participation rate									
80% participation	266 (4)	1 511 (16)	51 (2)	1 115 (9)	6 672 (133)	70.0 (0.1)	1 385 (33)	5 538 (104)	16 789 (362)
60% participation	214 (4)	1 255 (16)	38 (1)	843 (7)	5 366 (124)	60.8 (0.1)	1 091 (29)	5 170 (121)	16 131 (410)
Screening age									
40-65 every 2 years	306 (6)	1 700 (17)	90 (2)	1 276 (10)	8 570 (89)	79.7 (0.1)	1 557 (16)	5 458 (56)	18 714 (182)
40-70 every 2 years	353 (8)	1 962 (17)	94 (3)	1 588 (10)	9 287 (102)	92.0 (0.1)	1 637 (16)	6 150 (67)	19 432 (182)
50-70 every 2 years	274 (4)	1 509 (10)	40 (2)	1 201 (8)	6 196 (118)	73.6 (0.1)	1 529 (28)	6 259(116)	17 129 (313)
Screening interval									
45-70 every 3 years	262 (5)	1 465 (17)	45 (2)	1 649 (13)	6 411 (140)	64.9 (0.1)	1 331 (36)	4 990 (130)	15 284 (395)
40-65 every 3 years	253 (7)	1 422 (18)	65 (2)	1 525 (10)	6 966 (150)	65.3 (0.1)	1 252 (29)	4 656 (97)	16 385 (375)
40-70 every 3 years	286 (7)	1 627 (15)	67 (2)	1 894 (9)	7 474 (147)	74.2 (0.1)	1 308 (28)	5 257 (99)	17 013 (359)
50-70 every 3 years	209 (5)	1 224 (14)	29 (1)	1 311 (7)	4 931 (91)	59.6 (0.1)	1 221 (23)	5 009 (89)	14 437 (272)

Abbreviations: BC, Breast cancer; LYG, life years gained; ACER, average cost-effectiveness ratio. Note: All data expressed as mean (standard errors) per 100 000 women screened. ACERs are expressed as USD/LYG. Base scenario = screening women aged from 45-70 every 2 years. ^a^ Discounted at 5% for both LYG and costs.

###  Scenario Analysis

 The results for alternative scenarios are also shown in Table 3. Starting screening at a younger or older age (40 or 50 years) did not contribute to more discounted LYG compared to the base scenario. In addition, screening every 3 years was less effective than screening every 2 years, producing fewer averted BC deaths, screen-detected cancers and LYG.

**Figure 1:**
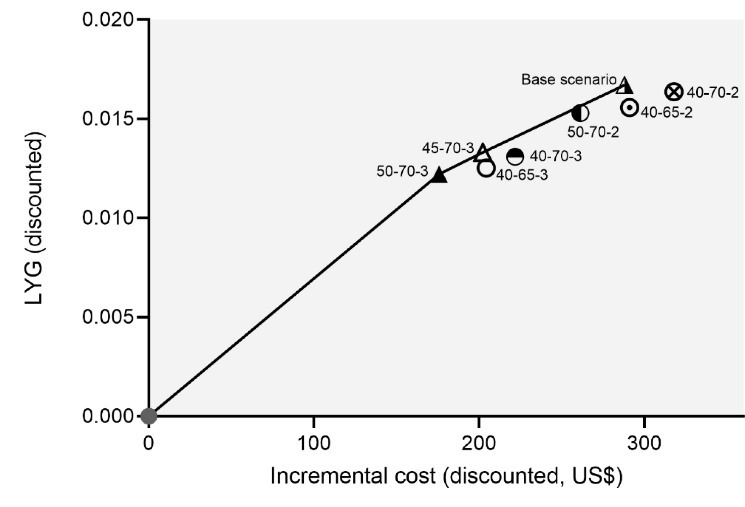
Cost-Effectiveness Efficient Frontier of BC Screening in Urban China. Note: Scenarios are expressed as screening starting age, stopping age and screening interval. Costs and LYGs were discounted at an annual rate of 5%. Abbreviations: BC, breast cancer; LYG, life years gained.

 The ICERs for the non-dominated scenarios were calculated and the efficient frontier is presented in Figure 1. The frontier consisted of three scenarios: 50-70 every 3 years scenario, 45-70 every 3 years scenario, and the base scenario, the corresponding ICERs were 14 437, 24 138, and 25 261 USD/LYG respectively. Using a threshold of 3 GDP per capita, the optimal scenario was the base scenario.

###  Sensitivity Analysis

**Figure 2:**
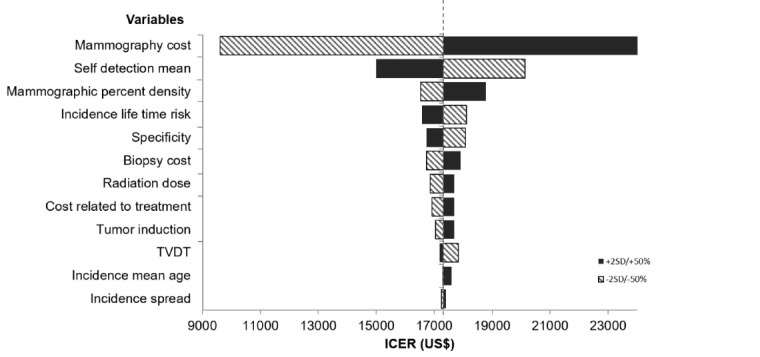
Univariate Sensitivity Analysis of Biennial Screening for Women Aged 45-70 Years in Urban China. Abbreviations: TVDT, tumour volume doubling time; ICER, incremental cost-effectiveness ratio.

 The sensitivity analysis is summarised in Figure 2. The base scenario remained cost-effective under the univariate sensitivity analysis. The ACERs for biennial screening of women aged 45-70 years were most sensitive to the mammography cost per screen, with discounted ACERs of 9605-24 981 USD/LYG, followed by mean self-detection size, and the percent mammographic density. The ACERs were moderately sensitive to TVDT, specificity, lifetime risk of BC, and other cost inputs (biopsy and treatment), and were least sensitive to the mean incidence age, and incidence standard deviation.

###  Budget Impact Analysis

 The budget impact analysis demonstrated that, if biennial screening was applied to a city with a population of 1 million, screening would incur a net cost for the healthcare system of almost 38.1 million USD over 10 years, of which 30.9 million USD would be due to the direct costs of screening tests and the remainder would be due to related biopsies and treatments (Table 4). When an 80% participation was assumed, the net cost decreased to 30.9 million USD.

**Table 4:** Result of Budget Impact Analysis

**Scenarios**	**Cost for Screening, USD Million**	**Cost for Biopsy and Treatment, USD Million**	**Total Net Cost, USD Million**
No screening	–	16.1	–
Base scenario, 100% participation	30.9	23.3	38.1
Base scenario, 80% participation	24.7	22.3	30.9

## Discussion

 We used a micro-simulation model to assess the benefit and cost-effectiveness of mammography screening in urban China. To get proper estimates, all input parameters were obtained based on systematic literature searches. In addition, the model was externally validated by comparing the simulation outcome with observed screening data, and showed that the performance of our model was generally acceptable. Specifically, the validation results showed that compared with observed data, the simulated CDR was slightly overestimated and the proportion of screen-detected tumours ≤2 cm was underestimated. The tumour size distributions of self-detected tumours were comparable between the data sets. Overall, the simulation indicated that biennial mammography screening for women aged 45-70 years is cost-effective in urban China with an ICER of 25 261 USD/LYG compared to the pervious efficient scenario. The model was most sensitive to self-detection size, followed by mammographic breast density and the cumulative lifetime risk of BC.

 Although mammography-based screening has proven effective in identifying cancers early, it is unavailable in many developing countries because of limited medical resources.^47^ Our results showed that at a 100% participation rate, screening 100 000 women aged 45-70 years yielded 7963 LYGs for an ACER of 17 309 USD/LYG. To date, there have been few cost-effectiveness studies of mammography as the main screening modality in China. Among this research, Wong et al reported a much higher discounted ICER of 42 500 USD/LYG when screening women aged 40–69 years compared to no screening,^48^ and Woo et al reported an ICER of 90 771 USD per disability-adjusted life-year (DALY) when screening women aged 50-74 years biennially.^49^ There are several reasons why the ICERs reported in their studies are less favourable. The main reason appears to be that these analyses were performed 10 years ago when costs for mammography, biopsy and treatments were higher. In addition, they included the indirect costs of time loss of patients and their family due to the treatment in their analyses. We also cannot ignore the fact that the incidence of BC has increased markedly during the last decade.^3^ Therefore, given that we used the latest data from the Chinese cancer registry, where the incidence rate is higher, it is not surprising that our data produced more favourable ICERs. In addition to biennial mammography screening, other strategies have been assessed elsewhere. For example, Sun et al,^50^ reported on screening of high-risk women annually by ultrasound or a combination of ultrasound and mammography depending on age. As expected, they reported a more favourable ICER of 8253 USD per quality-adjusted life-year (QALY). Nevertheless, direct comparison between these studies is difficult because of the different screening strategies and model designs.

 The SiMRiSc model has previously been validated and applied in a Western population.^14-17^ In the current study, we further adjusted and updated the input parameters of the model based on data for an Asian population. However, at present, population screening data from China are limited, so we opted to use data from Japan to validate our model. This revealed an acceptable fit of the CDR for BC screening, so we considered it acceptable for the purpose of this study. Nevertheless, compared with the observed data, the simulated CDR was higher and fewer screen-detected tumours ≤2 cm were identified. This could be explained by an imperfect referral rate for further diagnostic tests,^38^ with only 84.4% of screen-positives receiving a diagnostic test in their study, while we assumed that all screen-positives obtained a diagnostic test. Nevertheless, because the validation of our model showed a reasonably well result, we do not expect these slight deviations would change the major findings of our simulations.

 The simulation results showed that approximately 44% of cancers diagnosed during the screening period were interval cancers. Among those interval cancers, 48% interval cancers were true interval cancers, leaving the rest as cancers missed by the previous screening round. In a Dutch population, it was estimated that 39% of cancers diagnosed during screening period were interval cancers, which was lower than the estimation in our study (44%). This might be due to the biological characteristics of Chinese/Asian women, who tend to have smaller and denser breasts that lead to worse mammography performance and more interval cancers compared with Western cohorts.^51^ Few relevant studies have been conducted in Asia, but in a cohort from Singapore, approximately 34% of diagnosed cancers were interval cancers.^52^ However, those results should not be compared directly because they applied an older starting age (50 years) for screening. This is highly relevant because studies have shown that interval cancer rates in women aged 40-49 years were higher than in women aged ≥50 years, which could partly explain the larger proportion in our study.^14,53^ Nevertheless, it is more appropriate to compare our results with studies from urban China because the interval cancer rate can be influenced by the underlying incidence of BC, the definitions used and the age^53^; therefore, future studies are required with longer follow-up periods.

 The cost-effectiveness frontier showed that the base scenario recommended by the Chinese guideline was the most cost-effective scenario based on the discounted ICER (using a threshold of 3 GDP per capita). Regarding the age at which screening should start, scenarios starting from a younger or older age did not contribute to more discounted LYG compared to the base scenario. This may be due to the peak age of BC incidence in China, which is almost 10 years earlier than in Western countries.^54^ Overall, the results in this study indicated that a 25-year screening period from age 45-70 years is the optimal screening age for urban Chinese women.

 We performed a univariate sensitivity analysis by changing the base value to the lower and upper bounds of the 95% CI of model input parameter and by varying the costs by ±50%. This analysis indicated that our model was most sensitive to mammography cost per screen, and it also showed that even at 50% increased mammography cost per screen, the base scenario remained cost-effective. Other costs such as biopsy and treatment did not show large impacts on the uncertainty of our model. Parameters related to mammography performance, such as mammographic breast density and specificity, were also influential in the analysis, with a lower density and a higher specificity expected to result in a more favourable ICER. As shown in our univariate sensitivity analysis, the uncertainties of the input parameters only had a limited impact on the ACERs. Therefore, we did not perform a probabilistic sensitivity analysis as it was expected that the ACERs would be well below the threshold in most simulations.

 We used a time horizon of 10 years in the budget impact analysis because studies have shown that a follow-up of approximately 10 years is required before mortality reduction can be observed in an organized BC screening program.^55^ Regarding the population size, we used a population size of a medium-sized city in China, which is around 1 million. We expect that in a large country like China, it is likely that a population-based screening program will be introduced gradually, and our estimates based on a medium-sized city could provide practical information for policy-makers. In addition, as a meta-analysis showed that for a good-practice screening program, the attendance rate should be higher than 70% in Asian countries,^56^ we performed the budget impact analysis at a higher participation rate of 80%, which led to a net cost of 30.9 million USD compared to no screening. The budget impact analysis showed that net cost was mainly incurred by mammography examinations. Other related factors such as biopsy tests and treatment would also moderately increase the costs for the health-care payer.

 In our study, the health benefits were assessed using LYG instead of QALY. One of the main reasons why we used LYG instead of QALY is that there is scarce data reported health utility in screen-detected patients in the Chinese population. Second, in screening settings, the ultimate target of screening is to reduce mortality. Compared with QALY, LYG is a natural measure of that effect in a lifelong model as ours.^57^ In addition, several studies have shown that using LYG, DALY averted, or QALY did not lead to opposite decisions.^58,59^ In our study we also do not expect a major change in our outcomes when we would have used QALY instead of LYG because the ACER is far below the threshold of 3 times GDP per capita.

 Currently, there is no estimation of the expected attendance rate once a national BC program is implemented in urban China. Data from Japan showed that the attendance rate was surprisingly low at only 18.3%.^38^ In other Asian countries where population-based BC screening programs were implemented, such as Korea and Singapore, the attendance rates were 64% (in 2018) and 39% (in 2016), respectively.^60,61^ We anticipate that with improving awareness of BC and promotion measures by screening organizations, an attendance rate of 60%-80% in urban Chinese areas is likely to be achieved once a nationwide screening program is implemented.

 There are several important limitations to our study. First, ductal carcinoma in situ (DCIS) was not included in the model as crucial information on natural history and incidence in China is lacking. The detection of DCIS is a double-edged sword for screening effectiveness and cost-effectiveness. On the one hand, the detection and treatment of DCIS could prevent the development of invasive BCs and thus increase the screening effectiveness, but on the other hand, that also inflicts harms due to overdiagnosis and overtreatment. Whether the benefits of early detection of DCIS outweighs its harms will mainly depend on the aggressiveness of the treatment of DCIS and the percentage of DCISs that were overdiagnosed.^62^ On the whole, we do not expect that the addition of DCIS will profoundly alter our conclusion as less aggressive measures such as active monitoring for low-risk DCIS is likely to be applied,^62^ and as the extra cost due to the overdiagnosis and overtreatment of DCIS can be considered to be limited. Another limitation of not considering DCIS is that the number of overdiagnosed cancers was underestimated in our study as more DCIS will be detected with the introduction of population-based screening and as DCIS are more likely to be overdiagnosed than invasive cancers.^63^ Second, due to the paucity of studies considering BC screening in China, we needed to rely on data from studies in other Asian countries for model input and validation. However, we do not anticipate that these data will be markedly different from those for the urban Chinese population. Third, the SiMRiSc model validation was limited to detection rates and the tumour size distributions of screen-detected and self-detected tumours, and this necessitates that more studies are performed to assess other factors in biennial mammography screening, such as false positives and interval cancers. In addition, because a well-defined willingness-to-pay threshold specifically for LYG in China does not exist, a threshold of 3 times GDP per capita was used. This threshold was initially recommended when the health benefit was assessed by DALY averted.^44^ As the cost per LYG is likely higher than the cost per DALY averted,^57^ we anticipated that using the 3 times GDP threshold would be a fair one, and we expect that the main findings do not change if DALY would have been used. Also, because BC screening was only recently introduced in China, the mammographic specificity data that we used in our simulation may have been slightly overestimated, potentially resulting in an underestimation of the ICER. Despite this, the univariate sensitivity analysis showed that specificity only had a mild impact on the ICER. At a lower specificity of 90%, the ICER only increased by 5%, which does not significantly affect our conclusions.

## Conclusion

 As one of the most potent economies of the world, China has put ongoing effort to improve population health and to provide a better and equal health system in the country.^64^ To decrease the burden of disease posed by BC, the Chinese government recently introduced a biennial mammography screening strategy for women aged 45-70 years.^11^ At a threshold of triple the GDP, we showed that biennial mammography screening for women in this age interval is cost-effective in urban China when compared with no screening. Of note, the recommended screening age range from 45-70 years is appropriate because starting at a younger or an older age is less effective and less cost-effective. However, considering the large geographical and socioeconomic disparities across China, tailored screening strategies are required to further improve the effectiveness of BC screening among Chinese women.^65,66^

## Acknowledgements

 We thank Dr. Robert Sykes (https://www.doctored.org.uk) for providing editorial and language services. The author, J Wang, thanks the support from Chinese Scholarship Council for providing a scholarship for her PhD research.

## Ethical issues

 This article does not contain any studies with human participants or animals performed by any of the authors.

## Competing interests

 Authors declare that they have no competing interests.

## Authors’ contributions

 JW, SZ, and DWAvV searched the input files, JW, DWAvV, MJWG, WL, GHdB, and KMV contributed to the modification of the model, and interpretation of the results. JW, MJWG, GHdB, and WL contributed to the study design, formulating the research question, drafting and revising the manuscript. All authors have read the manuscript and approved for submission.

## Funding

 The study was funded by grants from the National Natural Science Foundation of China (grant number 81301799, and 72074166).

## Supplementary files


Supplementary file 1. Breast Cancer Survival Model.

Supplementary file 2. Breast Cancer Incidence Model.

Supplementary file 3. Age-Specific Mortality.

Supplementary file 4. Mammographic Sensitivity as a Function of Tumour Size.

Supplementary file 5. Meta-analysis for Mammography Specificity.

Supplementary file 6. Age Distribution in Urban China.

Supplementary file 7 contains Table S6.
