# Supplementary material for: Assessment of the Benefits and Cost-Effectiveness of Population-Based Breast Cancer Screening in Urban China: A Model-Based Analysis
Source: Int J Health Policy Manag. 2021 Jul 4;11(9):1658–67. doi: 10.34172/ijhpm.2021.62 (PMC9808213; doi:10.34172/ijhpm.2021.62)
Supplement: Supplementary file 1 — Breast Cancer Survival Model. [file ijhpm-11-1658-s001.pdf]

Article title: Assessment of the Benefits and Cost-Effectiveness of Population-Based Breast Cancer Screening in Urban China: A Model-Based Analysis

Journal name: International Journal of Health Policy and Management (IJHPM)

Authors' information: Jing Wang[1], Marcel J.W. Greuter[2,3], Senshuang Zheng[1], Daniëlle W.A. van Veldhuizen[1], Karin M. Vermeulen[1], Yuan Wang[4,5], Wenli Lu[4,5]*, Geertruida H. de Bock[1]

[1]Department of Epidemiology, University Medical Center Groningen, University of Groningen, Groningen, The Netherlands.

[2]Department of Radiology, University Medical Center Groningen, University of Groningen, Groningen, The Netherlands.

[3]Robotics and Mechatronics (RaM) Group, Faculty of Electrical Engineering Mathematics and Computer Science, Technical Medical Centre, University of Twente, Enschede, The Netherlands.

[4]Department of Epidemiology and Health Statistics, School of Public Health, Tianjin Medical University, Tianjin, China.

[5]Collaborative Innovation Center of Chronic Disease Prevention and Control, School of Public Health, Tianjin Medical University, Tianjin, China.

(*Corresponding author: luwenli@tmu.edu.cn)

Supplementary file 1. Breast Cancer Survival Model

The BC survival model was built based on a model presented in Michaelson et al. [1], where the correlation between tumor diameter (D) at diagnosis and survival F(D) described by the following equation:

$$F\ (D) = e^{-QD^{Z}}$$

In our analysis, we further expanded this model by adding another parameter namely time after diagnosis (T):

$$F\ (D,T) = e^{-Q(T)D^{Z(T)}}$$

Where Q and Z then can be made into continuous functions of *T:*

$$Q(T) = aT^b$$

$$Z(T) = c\ln T + d$$

As we did not find informative data from China, we used survival data from the Van Nuys Breast Center, USA [1] to obtain the related parameters values (a, b, c, d). Data from the Van Nuys showed that the 5-year and 10-year overall survival for breast cancer patients were 88%, and 77%, respectively, whereas a study from Shanghai, China reported that those rates were 93% and 83%, respectively [2]. Although the survival rates were higher in Shanghai, it is important to acknowledge that the survival rates for the whole urban China might be lower as better medical services can be appreciated in Shanghai than other cities. Thus, we considered the data from the Van Nuys breast cancers could be reliably used in our study. By fitting those data into the aforementioned functions, those parameters values were found to be:

Parameters	Value (SE)
a	$4.475*10^{-5}$($4.392*10^{-6}$)
b	1.859 (0.042)
c	-0.271 (0.101)
d	2.017 (0.037)

Reference:

1. Michaelson JS, Silverstein M, Wyatt J, et al. Predicting the survival of patients with breast carcinoma using tumor size. Cancer. 15;95:713-23. doi: 10.1002/cncr.10742.
2. Mo M, Yu J, Zhou C, et al. Changing long-term survival of Chinese breast cancer patients—experience from a large single institution hospital based cancer registry with 35 thousand patients. China Oncol. 2020;30:90-7.
