# Supplementary material for: Assessment of the Benefits and Cost-Effectiveness of Population-Based Breast Cancer Screening in Urban China: A Model-Based Analysis
Source: Int J Health Policy Manag. 2021 Jul 4;11(9):1658–67. doi: 10.34172/ijhpm.2021.62 (PMC9808213; doi:10.34172/ijhpm.2021.62)
Supplement: Supplementary file 2 — Breast Cancer Incidence Model. [file ijhpm-11-1658-s002.pdf]

Article title: Assessment of the Benefits and Cost-Effectiveness of Population-Based Breast Cancer Screening in Urban China: A Model-Based Analysis

Journal name: International Journal of Health Policy and Management (IJHPM)

Authors' information: Jing Wang[1], Marcel J.W. Greuter[2,3], Senshuang Zheng[1], Daniëlle W.A. van Veldhuizen[1], Karin M. Vermeulen[1], Yuan Wang[4,5], Wenli Lu[4,5]*, Geertruida H. de Bock[1]

[1]Department of Epidemiology, University Medical Center Groningen, University of Groningen, Groningen, The Netherlands.

[2]Department of Radiology, University Medical Center Groningen, University of Groningen, Groningen, The Netherlands.

[3]Robotics and Mechatronics (RaM) Group, Faculty of Electrical Engineering Mathematics and Computer Science, Technical Medical Centre, University of Twente, Enschede, The Netherlands.

[4]Department of Epidemiology and Health Statistics, School of Public Health, Tianjin Medical University, Tianjin, China.

[5]Collaborative Innovation Center of Chronic Disease Prevention and Control, School of Public Health, Tianjin Medical University, Tianjin, China.

(*Corresponding author: luwenli@tmu.edu.cn)

Supplementary file 2. Breast Cancer Incidence Model

In SiMRiSc model, breast cancer incidence was assumed to be normally-distributed as a function of age, allowing the formula can be expressed as:

$$p(a) = \frac{f}{\sigma\sqrt{2\pi}} e^{\frac{(\mu-a)^2}{2\sigma}} \quad \text{(Eq.[1])}$$

where p represents the risk of acquiring breast cancer during that age; a represents age; f represents lifetime risk; σ represents the standard deviation; μ represents mean age.

The related parameters can be deduced from the age-specific breast cancer incidence rates (Table S1).

Table S1 The age-specific incidence rate of invasive breast cancer in urban China

Age	Urban China (per 100 000)[1]	Japan (per 100 000)[2]
0 - 5	0.03	0.0
5 - 10	0.07	0.0
10 - 15	0.07	0.0
15 - 20	0.25	0.0
20 - 25	1.30	0.5
25 - 30	4.97	7.4
30 - 35	16.74	24.9
35 - 40	34.21	61.7
40 - 45	64.76	136.6
45 - 50	85.71	203.0
50 - 55	105.23	181.6
55 - 60	107.73	201.2
60 - 65	108.60	204.7
65 - 70	98.69	190.6
70 - 75	92.46	175.2
75 - 80	83.44	145.3
80-100	63.66	138.9

Data source:

1. He J, Chen WQ. Chinese cancer registry annual report 2019. Beijing: Press of Military Medical Sciences, pp204-205.
2. Cancer Information Service, National Cancer Center, Japan, 2011. Available from https://ganjoho.jp/en/professional/statistics/table_download.html
