# Supplementary material for: Assessment of the Benefits and Cost-Effectiveness of Population-Based Breast Cancer Screening in Urban China: A Model-Based Analysis
Source: Int J Health Policy Manag. 2021 Jul 4;11(9):1658–67. doi: 10.34172/ijhpm.2021.62 (PMC9808213; doi:10.34172/ijhpm.2021.62)
Supplement: Supplementary file 3 — Age-Specific Mortality. [file ijhpm-11-1658-s003.pdf]

Article title: Assessment of the Benefits and Cost-Effectiveness of Population-Based Breast Cancer Screening in Urban China: A Model-Based Analysis

Journal name: International Journal of Health Policy and Management (IJHPM)

Authors' information: Jing Wang[1], Marcel J.W. Greuter[2,3], Senshuang Zheng[1], Daniëlle W.A. van Veldhuizen[1], Karin M. Vermeulen[1], Yuan Wang[4,5], Wenli Lu[4,5]*, Geertruida H. de Bock[1]

[1]Department of Epidemiology, University Medical Center Groningen, University of Groningen, Groningen, The Netherlands.

[2]Department of Radiology, University Medical Center Groningen, University of Groningen, Groningen, The Netherlands.

[3]Robotics and Mechatronics (RaM) Group, Faculty of Electrical Engineering Mathematics and Computer Science, Technical Medical Centre, University of Twente, Enschede, The Netherlands.

[4]Department of Epidemiology and Health Statistics, School of Public Health, Tianjin Medical University, Tianjin, China.

[5]Collaborative Innovation Center of Chronic Disease Prevention and Control, School of Public Health, Tianjin Medical University, Tianjin, China.

(*Corresponding author: luwenli@tmu.edu.cn)

Supplementary file 3. Age-Specific Mortality

Table S2 The age-specific mortality rate in urban China, female*

Age group	N	Deaths	Mortality rate (‰)
0-4	535 681	403	0.75
5-9	536 240	83	0.15
10-14	504 878	73	0.14
15-19	539 356	97	0.18
20-24	745 762	125	0.17
25-29	985 224	210	0.21
30-34	779 090	242	0.31
35-39	738 736	387	0.52
40-44	893 151	715	0.80
45-49	945 689	1 186	1.25
50-54	798 231	1 420	1.78
55-59	587 589	1 942	3.31
60-64	608 154	3 077	5.06
65-69	427 382	3 762	8.80
70-74	289 243	4 574	15.81
75-79	218 980	6 470	29.55
80-84	145 324	7 569	52.08
85-89	69 912	5 777	82.63
90-94	23 410	3 061	130.76
95-99	4 765	880	184.71
100+	713	210	294.73

* Data source: China population and employment statistics yearbook 2016
