# Supplementary material for: Assessment of the Benefits and Cost-Effectiveness of Population-Based Breast Cancer Screening in Urban China: A Model-Based Analysis
Source: Int J Health Policy Manag. 2021 Jul 4;11(9):1658–67. doi: 10.34172/ijhpm.2021.62 (PMC9808213; doi:10.34172/ijhpm.2021.62)
Supplement: Supplementary file 4 — Mammographic Sensitivity as a Function of Tumour Size. [file ijhpm-11-1658-s004.pdf]

Article title: Assessment of the Benefits and Cost-Effectiveness of Population-Based Breast Cancer Screening in Urban China: A Model-Based Analysis

Journal name: International Journal of Health Policy and Management (IJHPM)

Authors' information: Jing Wang[1], Marcel J.W. Greuter[2,3], Senshuang Zheng[1], Daniëlle W.A. van Veldhuizen[1], Karin M. Vermeulen[1], Yuan Wang[4,5], Wenli Lu[4,5]*, Geertruida H. de Bock[1]

[1]Department of Epidemiology, University Medical Center Groningen, University of Groningen, Groningen, The Netherlands.

[2]Department of Radiology, University Medical Center Groningen, University of Groningen, Groningen, The Netherlands.

[3]Robotics and Mechatronics (RaM) Group, Faculty of Electrical Engineering Mathematics and Computer Science, Technical Medical Centre, University of Twente, Enschede, The Netherlands.

[4]Department of Epidemiology and Health Statistics, School of Public Health, Tianjin Medical University, Tianjin, China.

[5]Collaborative Innovation Center of Chronic Disease Prevention and Control, School of Public Health, Tianjin Medical University, Tianjin, China.

(*Corresponding author: luwenli@tmu.edu.cn)

Supplementary file 4. Mammographic Sensitivity as a Function of Tumour Size

The sensitivity function described in the paper by Isheden & Humphreys (2017) was applied in this study [19]. The sensitivity function was assumed as a logistic function (Figure S1), depending on both tumour diameter and percentage density:

$$Screening\ sensitivity\ (m, d)\ = \frac{1}{1+exp-\left(\beta_1+\beta_2 d+\beta_3 m+\beta_4 \frac{m}{d^2}\right)} \quad \text{(Eq. [2])}$$

This function consists of the variables diameter (d) and percentage density (m, scaled to [0,1]) and an interaction term m/d^2, which is included to capture an interplay between tumour size and area mammographic percent density (Isheden & Humphreys, 2017, Table S2). In addition, we also applied a systematic error of 0.1, which suggested that 10% of all cases that should be detected based on tumour volume would not be detected due to their characteristics such as lobular carcinomas, dense breast tissue and tumours located close to the thorax wall [14].

Table S3 Parameter estimates of the mammography sensitivity model

Parameters	Values (95%CI)
β_1	-4.38 (-3.76, -3.98)
β_2	0.49 (0.40, 0.60)
β_3	-1.34 (-3.00, -0.08)
β_4	-7.18 (-16.11, -2.77)
m	0.18 (0-0.43)

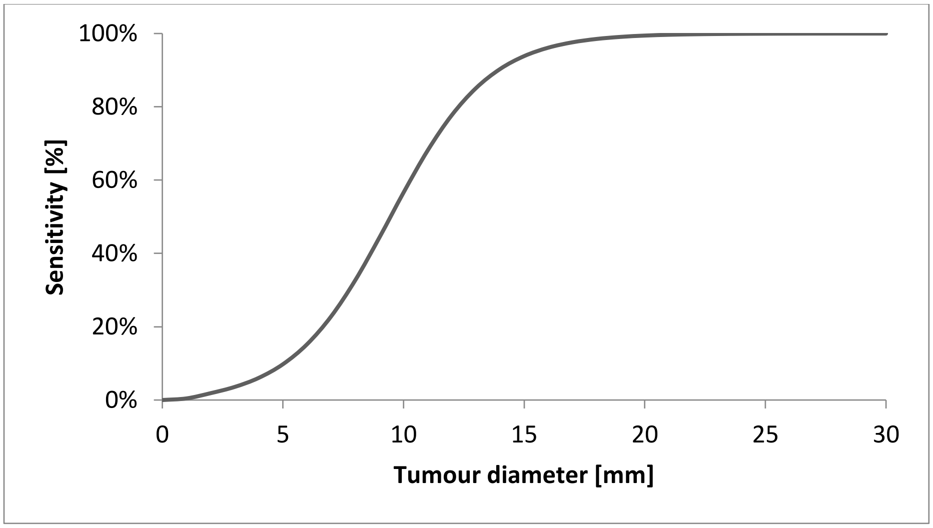

Figure S1 The mammography sensitivity as a function of tumour size
