# Supplementary material for: Assessment of the Benefits and Cost-Effectiveness of Population-Based Breast Cancer Screening in Urban China: A Model-Based Analysis
Source: Int J Health Policy Manag. 2021 Jul 4;11(9):1658–67. doi: 10.34172/ijhpm.2021.62 (PMC9808213; doi:10.34172/ijhpm.2021.62)
Supplement: Supplementary file 5 — Meta-analysis for Mammography Specificity. [file ijhpm-11-1658-s005.pdf]

Article title: Assessment of the Benefits and Cost-Effectiveness of Population-Based Breast Cancer Screening in Urban China: A Model-Based Analysis

Journal name: International Journal of Health Policy and Management (IJHPM)

Authors' information: Jing Wang[1], Marcel J.W. Greuter[2,3], Senshuang Zheng[1], Daniëlle W.A. van Veldhuizen[1], Karin M. Vermeulen[1], Yuan Wang[4,5], Wenli Lu[4,5]*, Geertruida H. de Bock[1]

[1]Department of Epidemiology, University Medical Center Groningen, University of Groningen, Groningen, The Netherlands.

[2]Department of Radiology, University Medical Center Groningen, University of Groningen, Groningen, The Netherlands.

[3]Robotics and Mechatronics (RaM) Group, Faculty of Electrical Engineering Mathematics and Computer Science, Technical Medical Centre, University of Twente, Enschede, The Netherlands.

[4]Department of Epidemiology and Health Statistics, School of Public Health, Tianjin Medical University, Tianjin, China.

[5]Collaborative Innovation Center of Chronic Disease Prevention and Control, School of Public Health, Tianjin Medical University, Tianjin, China.

(*Corresponding author: luwenli@tmu.edu.cn)

Supplementary file 5. Meta-analysis for Mammography Specificity

We performed a meta-analysis to investigate the performance of mammography in breast cancer screening in Asian women. A literature search was conducted for studies published between January 1, 2000, and December 31, 2019. PubMed, Web of science, and China National Knowledge Infrastructure databases were searched with the following keywords: "breast

neoplasms," "breast cancer," "mass screening", "radiography," "mammography," and "Asia." Study titles, abstracts, and texts were screened independently by two authors (J.W. and S.Zh.). A bivariate random model was used to estimate the pooled estimates of specificity with 95% confidence intervals (95% CI). Our search returned 2,689 English and 1,735 Chinese records, ten of which was included in the meta-analysis. Table S1 details the characteristics of the included studies.

Table S4. Characteristics of Included Studies of Breast Cancer Screening

First author[ref.]	Year of Publication	Study Design	Country	Starting age	Risk Level	Follow-up (Year)	Sample Size	True Positive Number (%)	False Positive Number (%)	False Negative Number (%)	True Negative Number (%)
H Dong[9]	2018	Cohort	China	45	Average	1.0	31918	84(0.3)	604(1.9)	15(0.1)	31215(97.7)
C Chou[26]	2012	Cohort	China	50	Average	NA	184580	767(0.4)	18705(10.1)	151(0.1)	164957(89.4)
M Kang[27]	2014	Cohort	China	35	Average	1.0	2471	11(0.5)	24(1.0)	3(0.1)	2433(98.4)
S Honjo[28]	2007	Cohort	Japan	30	Average	1.0	3453	8(0.2)	271(7.8)	5(0.2)	3169(91.8)
K Ohta[29]	2014	Cohort	Japan	40	Average	0.5	55086	154(0.3)	5872(10.7)	55(0.1)	49005(88.9)
C Chen[30]	2008	Cohort	China	40	Average	0.5	8249	22(0.3)	685(8.3)	5(0.1)	7537(91.3)
M Kang[31]	2010	Cohort	China	30	Average	1.0	8718	32(0.4)	79(0.9)	4(0.1)	8603(98.6)
H Moon[32]	2015	Cohort	Korea	40	Average	1.0	4394	13(0.3)	486(11.1)	4(0.1)	3891(88.5)
E Lee[33]	2016	Cohort	Korea	40	Average	1.0	128756	346(0.3)	24259(18.8)	54(0.0)	104097(80.5)
A Suzuki[34]	2008	Cohort	Japan	40	Average	2.0	112071	289(0.3)	9779(8.6)	56(0.1)	101947(91.0)

Abbreviations: NA=Not applicable.
