# Supplementary material for: Assessment of the Benefits and Cost-Effectiveness of Population-Based Breast Cancer Screening in Urban China: A Model-Based Analysis
Source: Int J Health Policy Manag. 2021 Jul 4;11(9):1658–67. doi: 10.34172/ijhpm.2021.62 (PMC9808213; doi:10.34172/ijhpm.2021.62)
Supplement: Supplementary file 6 — Age Distribution in Urban China. [file ijhpm-11-1658-s006.pdf]

Article title: Assessment of the Benefits and Cost-Effectiveness of Population-Based Breast Cancer Screening in Urban China: A Model-Based Analysis

Journal name: International Journal of Health Policy and Management (IJHPM)

Authors' information: Jing Wang[1], Marcel J.W. Greuter[2,3], Senshuang Zheng[1], Daniëlle W.A. van Veldhuizen[1], Karin M. Vermeulen[1], Yuan Wang[4,5], Wenli Lu[4,5]*, Geertruida H. de Bock[1]

[1]Department of Epidemiology, University Medical Center Groningen, University of Groningen, Groningen, The Netherlands.

[2]Department of Radiology, University Medical Center Groningen, University of Groningen, Groningen, The Netherlands.

[3]Robotics and Mechatronics (RaM) Group, Faculty of Electrical Engineering Mathematics and Computer Science, Technical Medical Centre, University of Twente, Enschede, The Netherlands.

[4]Department of Epidemiology and Health Statistics, School of Public Health, Tianjin Medical University, Tianjin, China.

[5]Collaborative Innovation Center of Chronic Disease Prevention and Control, School of Public Health, Tianjin Medical University, Tianjin, China.

(*Corresponding author: luwenli@tmu.edu.cn)

Supplementary file 6. Age Distribution in Urban China

Table S5 The age distribution in urban China, 2017*

Age	Percentage (%)
New eligible women (every year)	0.75
45-49	4.50
50-54	4.20
55-59	2.58
60-64	2.97
65-69	2.29

*Data source: National Bureau of Statistics of China Internet.
