# Supplementary material for: Assessment of the Benefits and Cost-Effectiveness of Population-Based Breast Cancer Screening in Urban China: A Model-Based Analysis
Source: Int J Health Policy Manag. 2021 Jul 4;11(9):1658–67. doi: 10.34172/ijhpm.2021.62 (PMC9808213; doi:10.34172/ijhpm.2021.62)
Supplement: Supplementary file 7 — contains Table S6. [file ijhpm-11-1658-s007.pdf]

Article title: Assessment of the Benefits and Cost-Effectiveness of Population-Based Breast Cancer Screening in Urban China: A Model-Based Analysis

Journal name: International Journal of Health Policy and Management (IJHPM)

Authors' information: Jing Wang[1], Marcel J.W. Greuter[2,3], Senshuang Zheng[1], Daniëlle W.A. van Veldhuizen[1], Karin M. Vermeulen[1], Yuan Wang[4,5], Wenli Lu[4,5]*, Geertruida H. de Bock[1]

[1]Department of Epidemiology, University Medical Center Groningen, University of Groningen, Groningen, The Netherlands.

[2]Department of Radiology, University Medical Center Groningen, University of Groningen, Groningen, The Netherlands.

[3]Robotics and Mechatronics (RaM) Group, Faculty of Electrical Engineering Mathematics and Computer Science, Technical Medical Centre, University of Twente, Enschede, The Netherlands.

[4]Department of Epidemiology and Health Statistics, School of Public Health, Tianjin Medical University, Tianjin, China.

[5]Collaborative Innovation Center of Chronic Disease Prevention and Control, School of Public Health, Tianjin Medical University, Tianjin, China.

(*Corresponding author: luwenli@tmu.edu.cn)

Supplementary file 7

Table S6. Additional information on outcomes of the SiMRiSc model for breast cancer screening in China

Scenario	BC diagnosed from age 45 onwards*(SE)	Total number of BC deaths (SE)	Number of false positives (SE)
No screening	3 614 (17)	2 118 (17)	
Base scenario, 100% attendance rate	3 654 (17)	1 805 (16)	75 110 (128)
Base scenario, 80% attendance rate	3 639 (17)	1 852 (17)	60 081 (99)
Base scenario, 60% attendance rate	3 627 (17)	1 904 (17)	45 054 (77)

Note: All numbers are given per 100,000 women screened with SE. BC= Breast cancer.
*Both screen- and self-detected tumors are counted.
